# Inactivation of Severe Acute Respiratory Syndrome Coronavirus 2 by WHO-Recommended Hand Rub Formulations and Alcohols

**DOI:** 10.3201/eid2607.200915

**Published:** 2020-07

**Authors:** Annika Kratzel, Daniel Todt, Philip V’kovski, Silvio Steiner, Mitra Gultom, Tran Thi Nhu Thao, Nadine Ebert, Melle Holwerda, Jörg Steinmann, Daniela Niemeyer, Ronald Dijkman, Günter Kampf, Christian Drosten, Eike Steinmann, Volker Thiel, Stephanie Pfaender

**Affiliations:** Institute of Virology and Immunology, Bern and Mittelhäusern, Switzerland (A. Kratzel, P. V’kovski, S. Steiner, M. Gultom, T.T.N. Thao, N. Ebert, M. Holwerda, R. Dijkman, V. Thiel);; University of Bern, Bern (A. Kratzel, P. V’kovski, S. Steiner, M. Gultom, T.T.N. Thao, N. Ebert, M. Holwerda, R. Dijkman, V. Thiel);; Ruhr University Bochum, Germany (D. Todt, E. Steinmann, S. Pfaender);; Institute of Medical Microbiology, University Hospital of Essen, Essen, Germany (J. Steinmann);; Institute of Clinical Hygiene, Medical Microbiology and Infectiology General Hospital Nürnberg, Paracelsus Medical University, Nürnberg, Germany (J. Steinmann);; Institute of Virology, Charité Berlin, Berlin, Germany (D. Niemeyer, C. Drosten);; Institute for Hygiene and Environmental Medicine, University Medicine Greifswald, Greifswald, Germany (G. Kampf)

**Keywords:** ethanol, 2-propanol, hand sanitizer, World Health Organization, coronavirus disease, respiratory infections, severe acute respiratory syndrome coronavirus 2, SARS-CoV-2, SARS, COVID-19, 2019 novel coronavirus disease, coronavirus disease, zoonoses, viruses, coronavirus

## Abstract

Infection control instructions call for use of alcohol-based hand rub solutions to inactivate severe acute respiratory syndrome coronavirus 2. We determined the virucidal activity of World Health Organization–recommended hand rub formulations, at full strength and multiple dilutions, and of the active ingredients. All disinfectants demonstrated efficient virus inactivation.

Severe acute respiratory syndrome coronavirus 2 (SARS-CoV-2) is the third highly pathogenic human coronavirus to cross the species barrier into the human population during the past 20 years (*1*–*3*). SARS-CoV-2 infection is associated with coronavirus disease (COVID-19), which is characterized by severe respiratory distress, fever, and cough and high rates of mortality, especially in older persons and those with underlying health conditions (*3*). The World Health Organization (WHO) declared SARS-CoV-2 a pandemic on March 11, 2020 (*4*), and by April 8, a total of 1,447,466 confirmed cases and 83,471 deaths from SARS-CoV-2 had been reported worldwide (*5*). 

Human-to-human transmission of SARS-CoV-2 is efficient, and infected persons can transmit the virus even when they have no, or only mild, symptoms (*3*). Because no antiviral drugs or vaccines are available, virus containment and prevention of infection are the current highest priorities. To limit virus spread, effective hand hygiene is crucial. Therefore, easily available but efficient disinfectants are needed. WHO’s guidelines for hand hygiene in healthcare suggest 2 alcohol-based formulations for hand sanitization to reduce the infectivity and spread of pathogens (*6*). WHO’s recommendations are based on fast-acting, broad-spectrum microbicidal activity, along with accessibility and safety. The original WHO formulations failed to meet the efficacy requirements of European Norm 1500 in previous tests (*7*). However, Suchomel et al. (*8*) suggested modified versions with increased concentrations of ethanol: 80% (wt/wt) (85.5% [vol/vol]; formulation I), or isopropanol, 75% (wt/wt) (81.3% [vol/vol]; formulations II). Later, they complemented these by reducing the glycerol concentrations (*9*). 

We previously showed that these modified WHO formulations were able to inactivate severe acute respiratory syndrome coronavirus (SARS-CoV) and Middle East respiratory syndrome coronavirus (MERS-CoV; *10*), which are related to SARS-CoV-2. Current recommendations to inactivate SARS-CoV-2 were translated from findings of other coronaviruses (*11*). To evaluate whether these alcohol-based disinfectants also effectively inactivate SARS-CoV-2, we tested different concentrations of the original and modified WHO formulations I and II (*6*,*9*), ethanol, and 2-propanol for virucidal activity.

## The Study

We propagated SARS-CoV-2 (SARS-CoV-2/München-1.1/2020/929) on VeroE6 cells (kindly provided by M. Müller and C. Drosten; Charité, Berlin, Germany). We cultured VeroE6 cells in Dulbecco’s modified minimal essential medium supplemented with 10% heat inactivated fetal bovine serum, 1% nonessential amino acids, 100 µg/mL of streptomycin and 100 IU/mL of penicillin, and 15 mMol of HEPES (Gibco; ThermoFisher, https://www.thermofisher.com).

Original WHO formulation I consists of 80% (vol/vol) ethanol, 1.45% (vol/vol) glycerol, and 0.125% (vol/vol) hydrogen peroxide. Original WHO formulation II consists of 75% (vol/vol) 2-propanol, 1.45% (vol/vol) glycerol, and 0.125% (vol/vol) hydrogen peroxide. The modified WHO formulation I used in our study consists of 80% (wt/wt) ethanol, 0.725% (vol/vol) glycerol, and 0.125% (vol/vol) hydrogen peroxide. The modified isopropyl-based WHO formulation II contains 75% (wt/wt) 2-propanol, 0.725% (vol/vol) glycerol, and 0.125% (vol/vol) hydrogen peroxide (*9*). We also prepared ethanol (CAS 64–17–5) and 2-propanol (CAS 67–63–0) in vol/vol dilutions for investigation.

We performed virucidal activity studies by using a quantitative suspension test with 30 s exposure time (*6*). In brief, we mixed 1 part virus suspension with 1 part organic load (0.3% bovine serum albumin as an interfering substance) and 8 parts disinfectant solution of different concentrations. After a 30 s exposure, we serially diluted samples and determined the 50% tissue culture infectious dose (TCID_50_) per milliliter by using crystal violet staining and subsequently scoring the number of wells displaying cytopathic effects. We calculated TCID_50_ by the Spearman-Kärber algorithm, as described (*12*). We monitored the cytotoxic effects of disinfectants by using crystal violet staining and optical analysis for altered density and morphology of the cellular monolayer in the absence of virus. We quantified cytotoxic effects analogous to the TCID_50_/mL of the virus infectivity.

**Figure 3:**
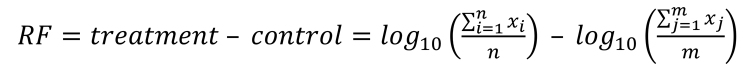
Formula

We determined dose-response curves as percent normalized virus inactivation versus percent log disinfectant concentration by nonlinear regression using the robust fitting method on the normalized TCID_50_ data implemented in Prism version 8.0.3 (GraphPad, https://www.graphpad.com). We plotted reference curves for SARS-CoV, MERS-CoV, and bovine CoV (BCoV) by using previously published data (*9*). BCoV is often used as surrogate for highly pathogenic human CoVs. We assessed the mean TCID_50_ and standard deviations of means from 3 individual experiments. We identified outliers by using Grubb’s test in Prism. We calculated reduction factors (RFs) for each treatment condition as follows (Figure 3).

**Figure 1:**
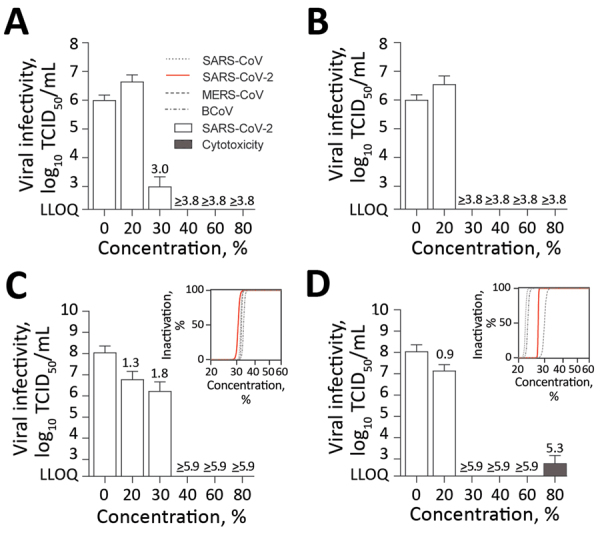
Virucidal activity of original and modified World Health Organization (WHO)–recommended hand rub formulations I and II for inactivating severe acute respiratory syndrome coronavirus 2 (SARS-CoV-2). The means of 3 independent experiments with SDs (error bars) and percentage of inactivation at different concentrations are shown. A) WHO original formulation I; B) WHO original formulation II; C) WHO modified formulation I; D) WHO modified formulation II. Insets in panels C and D show regression analyses of the inactivation of coronaviruses. Dark gray bar shows cytotoxic effects, calculated analogous to virus infectivity. Reduction factors are included above the bar. Dilutions of the WHO formulations ranged from 0–80% with an exposure time of 30 s. Viral titers are displayed as TCID_50_/mL values. BCoV, bovine coronavirus; LLOQ, lower limit of quantification; MERS-CoV, Middle East respiratory syndrome coronavirus; SARS-CoV, severe acute respiratory syndrome coronavirus; SARS-CoV-2, severe acute respiratory syndrome coronavirus 2; TCID_50_/mL, 50% tissue culture infectious dose.

 Our results showed that SARS-CoV-2 was highly susceptible to the original and the modified WHO formulations (Figure 1). The original and modified versions of formulation I efficiently inactivated the virus. The original formulation I of 80% (vol/vol) ethanol had an RF of >3.8 (Figure 1, panel A) and the modified formulation I of 80% (wt/wt) ethanol had an RF of >5.9 (Figure 1, panel C). Dilutions >40% were still effective (Figure 1, panels A and C). Subsequent regression analysis of modified formulation I revealed similar inactivation profiles compared with SARS-CoV, MERS-CoV, and BCoV. (Figure 1, panel C). The original and modified versions of formulation II also were effective. The original formulation II of 75% (vol/vol) 2-propanol had a log_10_-reduction of >3.8 (Figure 1, panel B) and the modified formulation II of 75% (wt/wt) 2-propanol had a log_10_-reduction of >5.9. Dilution >30% (vol/vol) also resulted in complete viral inactivation (Figure 1, panel D). Regression analysis of modified WHO formulation II showed the inactivation profile of SARS-CoV-2 was comparable to those of SARS-CoV, BCoV, and MERS-CoV **(**Figure 1, panel D**)**.

**Figure 2:**
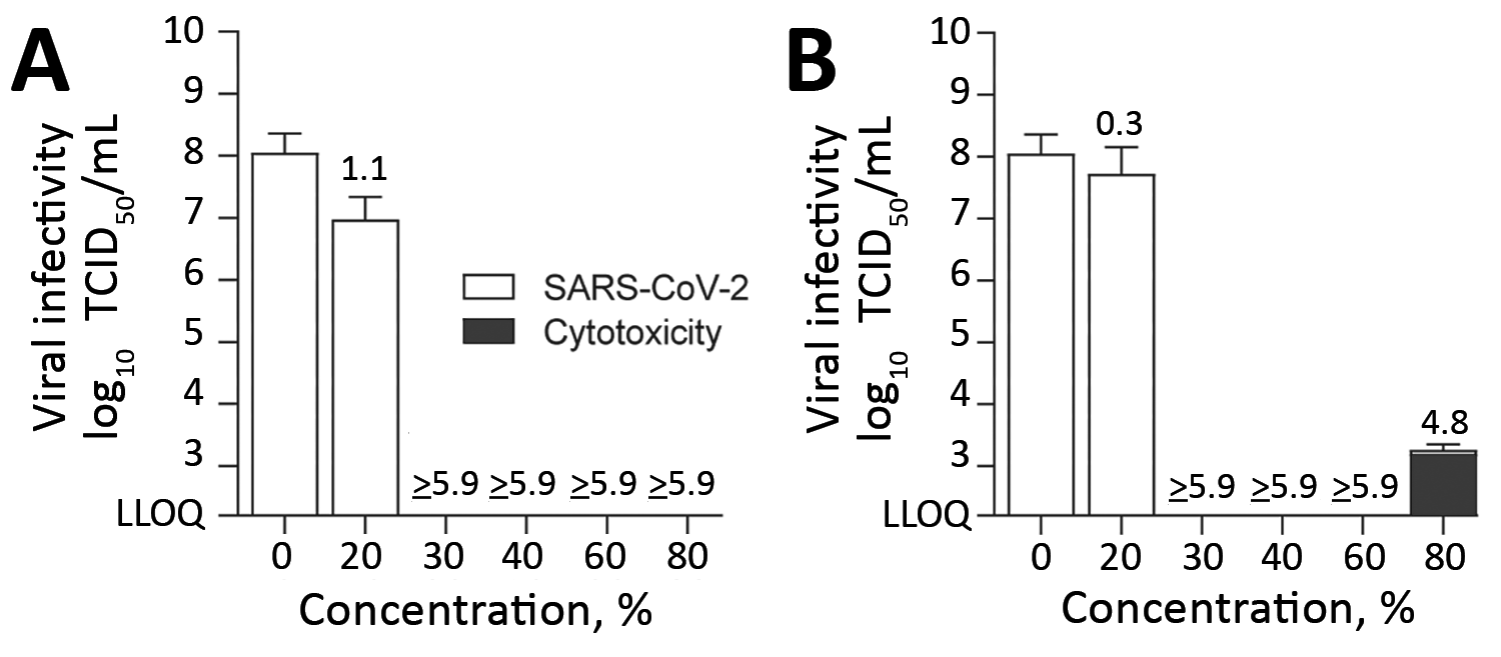
Effect of commercially available alcohols in inactivating SARS-CoV-2. The means of 3 independent experiments with SDs (error bars) are shown. A) Results for ethanol. B) Results for 2-propanol. Dark gray bar indicates cytotoxic effects, calculated analogous to virus infectivity. Reduction factors are included above the bar. The biocide concentrations ranged from 0–80% with an exposure time of 30 s. Viral titers are displayed as TCID_50_/mL values. LLOQ, lower limit of quantification; SARS-CoV-2, severe acute respiratory syndrome coronavirus 2; TCID_50_/mL, 50% tissue culture infectious dose.

We also investigated the susceptibility of SARS-CoV-2 against the active components of the WHO-recommended formulations, which are also the active ingredients of commercially available hand disinfectants. Ethanol (Figure 2, panel A) and 2-propanol (Figure 2, panel B) were able to reduce viral titers to background levels in 30 s with RFs of between 4.8 and ≥5.9. Furthermore, we noted that a concentration of >30% (vol/vol) ethanol or 2-propanol is sufficient for complete viral inactivation (Figure 2).

## Conclusions

We found that SARS-CoV-2 was efficiently inactivated by WHO-recommended formulations, supporting their use in healthcare systems and viral outbreaks. Of note, both the original and modified formulations were able to reduce viral titers to background level within 30 s. In addition, ethanol and 2-propanol were efficient in inactivating the virus in 30 s at a concentration of >30% (vol/vol). Alcohol constitutes the basis for many hand rubs routinely used in healthcare settings. One caveat of this study is the defined inactivation time of exactly 30 s, which is the time recommended but not routinely performed in practice. Our findings are crucial to minimize viral transmission and maximize virus inactivation in the current SARS-CoV-2 outbreak.
